# Measuring functional disability in children with developmental disorders in low-resource settings: validation of Developmental Disorders-Children Disability Assessment Schedule (DD-CDAS) in rural Pakistan

**DOI:** 10.1017/gmh.2020.13

**Published:** 2020-08-03

**Authors:** Syed Usman Hamdani, Zill-e Huma, Lawrence Wissow, Atif Rahman, Melissa Gladstone

The author apologises that upon publication, three figures were transposed and shown placed in the wrong order within the article.

**Fig. 3.**
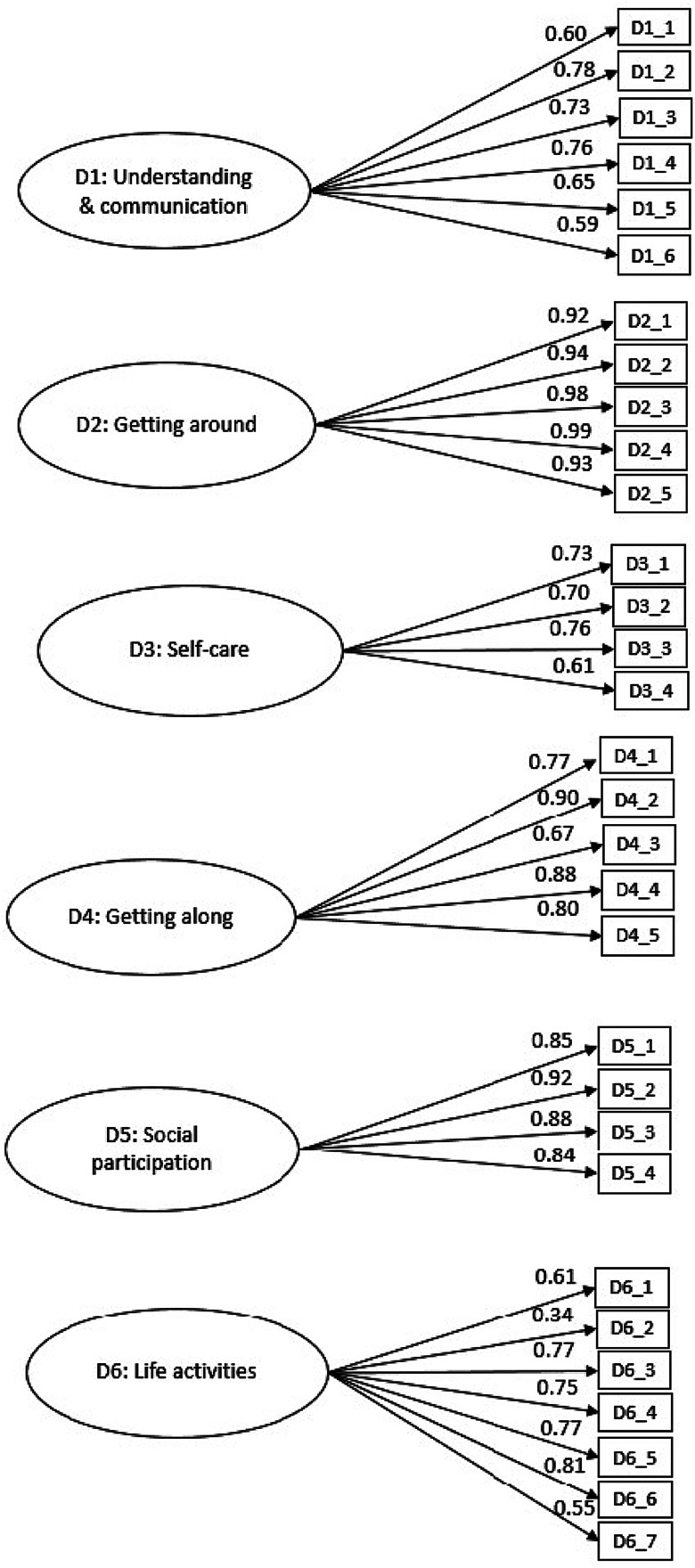
First-order confirmatory factor analysis of the Developmental Disorders-Children Disability Assessment Schedule (DD-CDAS).

**Fig. 4.**
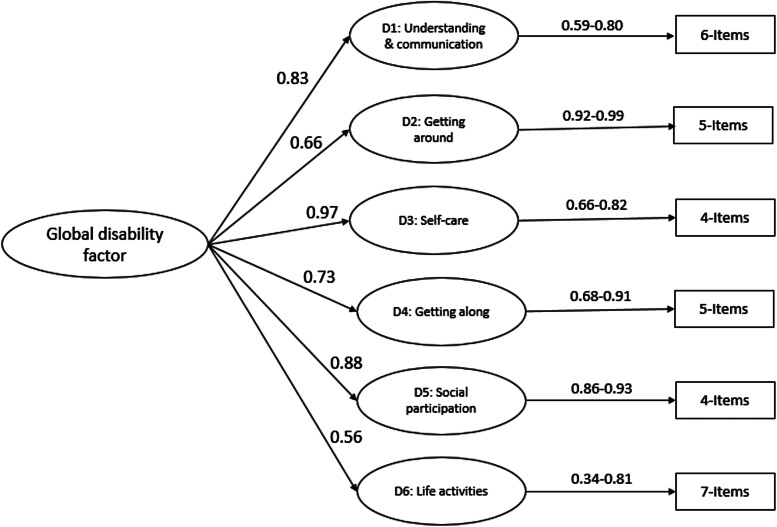
Second-order confirmatory factor analysis of Developmental Disorders-Children Disability Assessment Schedule for Developmental disorders (DD-CDAS).

Figure 4 in the article should have been Figure 3

**Fig. 5.**
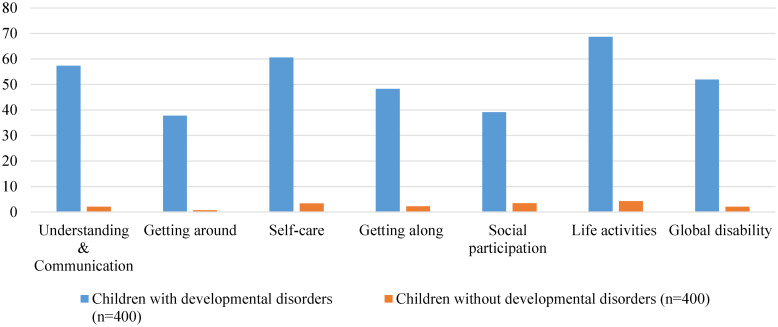
Discriminative validity of DD-CDAS (*N* = 800). DD-CDAS, Developmental Disorders-Children Disability Assessment Schedule.

The existing Figure 5 in the article should have been Figure 4

And Figure 3 in the article should have been Figure 5:
